# Dynamically Shaping Chaperones. Allosteric Modulators of HSP90 Family as Regulatory Tools of Cell Metabolism in Neoplastic Progression

**DOI:** 10.3389/fonc.2020.01177

**Published:** 2020-07-16

**Authors:** Carlos Sanchez-Martin, Stefano A. Serapian, Giorgio Colombo, Andrea Rasola

**Affiliations:** ^1^Dipartimento di Scienze Biomediche, Università di Padova, Padua, Italy; ^2^Dipartimento di Chimica, Università di Pavia, Pavia, Italy; ^3^Istituto di Chimica del Riconoscimento Molecolare, CNR, Milan, Italy

**Keywords:** chaperones, HSP90, TRAP1, mitochondria, tumor metabolism, allosteric inhibitor, ATP-competitive inhibitors, anti-neoplastic strategies

## Abstract

Molecular chaperones have recently emerged as fundamental regulators of salient biological routines, including metabolic adaptations to environmental changes. Yet, many of the molecular mechanisms at the basis of their functions are still unknown or at least uncertain. This is in part due to the lack of chemical tools that can interact with the chaperones to induce measurable functional perturbations. In this context, the use of small molecules as modulators of protein functions has proven relevant for the investigation of a number of biomolecular systems. Herein, we focus on the functions, interactions and signaling pathways of the HSP90 family of molecular chaperones as possible targets for the discovery of new molecular entities aimed at tuning their activity and interactions. HSP90 and its mitochondrial paralog, TRAP1, regulate the activity of crucial metabolic circuitries, making cells capable of efficiently using available energy sources, with relevant implications both in healthy conditions and in a variety of disease states and especially cancer. The design of small-molecules targeting the chaperone cycle of HSP90 and able to inhibit or stimulate the activity of the protein can provide opportunities to finely dissect their biochemical activities and to obtain lead compounds to develop novel, mechanism-based drugs.

## Introduction

Chaperones are molecular machines that assist folding, conformational changes and subcellular trafficking of proteins and control their degradation following aggregation, unfolding or misfolding. Fine and orchestrated tuning of these processes is carried out by different chaperone families and leads to maintenance and quality control of the proteome, an extremely complex and vital task for cells (1). Heat Shock Protein 90 (HSP90) proteins are chaperones that exert their regulatory functions on the structure and activity of a variety of diverse client proteins, thus integrating signaling and metabolic circuitries and acting as crucial components in consenting flexible adaptations of cells to environmental changes and stresses (2).

Exposure to harmful environmental stimuli is a common event in the process of tumor growth. Fluctuations in pH, oxygen, or nutrient availability prompt a profound rewiring of the metabolic circuitries of neoplastic cells and major changes in the homeostasis of their proteome (proteostasis) (3, 4). These noxious conditions also affect biochemical functions confined in specific subcellular compartments, such as protein folding in the endoplasmic reticulum (ER) (5, 6) as well as the bioenergetic functions of mitochondria (7, 8). Activation of organelle-restricted signaling pathways and metabolic adaptations can subtly regulate the equilibrium among death, dormancy, and aggressiveness of tumor cells (9–11). In order to cope with these stresses and sustain pro-oncogenic biological routines, including growth, proliferation, invasion, metastasis and evasion from death stimuli, most cancer cells overexpress HSP90 family members (12, 13). The various paralogs of HSP90 proteins can play a key role at the crossroads of these multiple cellular functions in the different cellular districts, namely HSP90 in the cytosol, Grp94 in the ER and TRAP1 in mitochondria (14–16); extracellular HSP90 is also involved in cell-to-cell communication (17). Induction of Hsp90 family protein expression contributes to the adaptations of the metabolic machineries in tumor cells and has been associated with cancer progression, resistance to chemotherapy and poor prognosis (12, 18). Adaptability to stress conditions is linked to specific subsets of clients, and the range of functional flexibility of the chaperone and of potential activities of its interactors are further expanded both by post-translational modifications and by the recruitment of other chaperones and of co-chaperones (19–21). In this context, the development of drugs targeting HSP90 components has emerged as a promising anti-neoplastic strategy.

Here, we report our views on molecular design strategies aimed to act on the circuitries in which HSP90 family members play a key role in cancer cells. In particular, we focus on cytosolic HSP90 and its mitochondrial paralog TRAP1, as Grp94 has a more specialized role in the maturation process of particular secretory and membrane-bound proteins clients, such as immunoglobulins, integrins, and Toll-like receptors (14). We will discuss interventions that range from the use of allosteric modulators of chaperone functions, to the targeting of protein-protein interactions involved in the assembly of functional complexes. We also discuss possible perspectives in combining the use of molecules that target HSP90 complexes with the use of other antineoplastic compounds, with a particular focus on the control of metabolic vulnerabilities in cancer models.

## Structure and Function of HSP90 Molecular Chaperones

**Figure 1:**
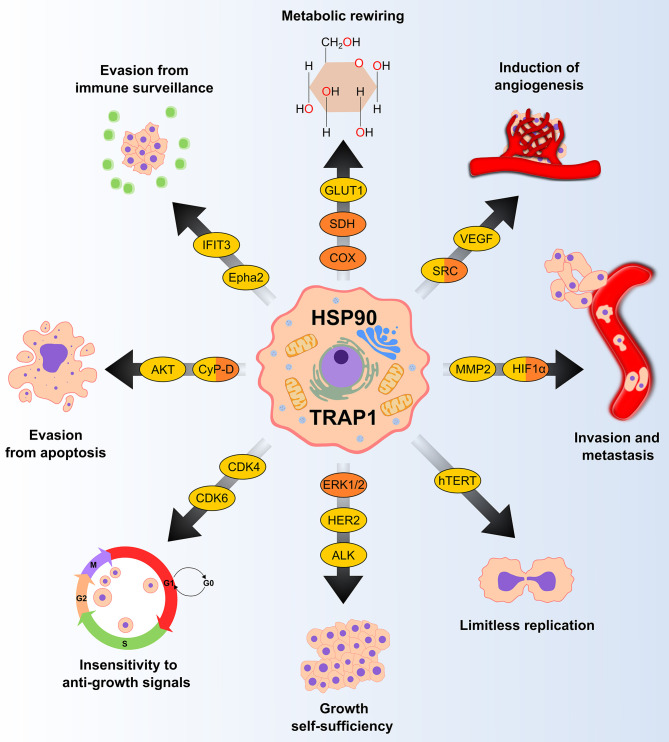
HSP90 and TRAP1 chaperones are involved in several pro-neoplastic biological processes. Many clients of HSP90 and TRAP1 (in yellow and orange, respectively) are proteins that regulate key tumorigenic processes. GLUT1, glucose transporter 1; SDH, succinate dehydrogenase; COX, cytochrome *c* oxidase; VEGF, vascular endothelial growth factor; MMP2, matrix metallopeptidase 2; HIF1α, hypoxia-inducible factor 1-alpha; hTERT, human telomerase reverse transcriptase; ERK 1/2, extracellular signal-regulated kinase 1/2; HER2, human epidermal growth factor receptor 2; ALK, anaplastic lymphoma kinase; CDK, cyclin-dependent kinase; CyP-D, cyclophilin D; Epha2, ephrin type-A receptor 2; IFIT3; Interferon induced protein with tetratricopeptide repeats 3.

Molecular chaperones of the HSP90 family are essential cell constituents, making up 1–2% of the proteome. Their expression can be further stimulated by stress, and in tumor cells they can reach up to the 4–7% of the expressed proteome, thus shaping all biological processes required for neoplastic progression (Figure 1). HSP90 exists as a homodimer and each individual chain consists of three globular domains (22). Structures reporting different full length chaperone isoforms can be found at the following pdb codes: 2ioq, 2iop, 2cg9, 2o1v, 2o1u, 4job, 4ipe, 4iyn, 5uls, 5tvu, 5tvx, 6d14, 5tth, 5tvw.

The structures show the common organization in a N-terminal ATP-binding domain (N-domain), a middle domain (M-domain) involved in ATP hydrolysis, and a C-terminal domain (C-domain) responsible for HSP90 dimerization and for interactions with several co-chaperones. HSP90, TRAP1, and Grp94 have a mutual sequence identity of about 30–40%, which reflects in the high structural similarity and alignability of their individual domains (23–25). However, the preferential relative orientation of the domains in the crystal structures solved so far varies significantly depending on the protein, cellular compartment, and organism (26), yielding a global root mean square deviation (RMSD) of atomic positions of at least 7Å.

HSP90 chaperones manifest their functions by promoting the folding and tuning the activity of a plethora of clients endowed with highly diverse structures, cellular localizations and functions. The two main cytosolic HSP90 isoforms, HSP90α and HSP90β, have an interactome that includes more than 400 putative clients (https://www.picard.ch/HSP90Int/index.php), making them central modulators of at least a dozen of important biochemical pathways, including stress regulation, protein folding, DNA repair, kinase signaling, cell survival and metabolism (2, 12). HSP90 effects on clients encompass facilitating the formation of specific protein conformations, as in the case of kinase activation (27), prompting the assembly of multiprotein complexes (28), stabilizing the binding-competent conformation of ligand receptors, and regulating protein dynamics and conformational state ensembles (29). Client stability depends on the chaperone, and its inhibition induces proteasomal degradation of client proteins.

**Figure 2:**
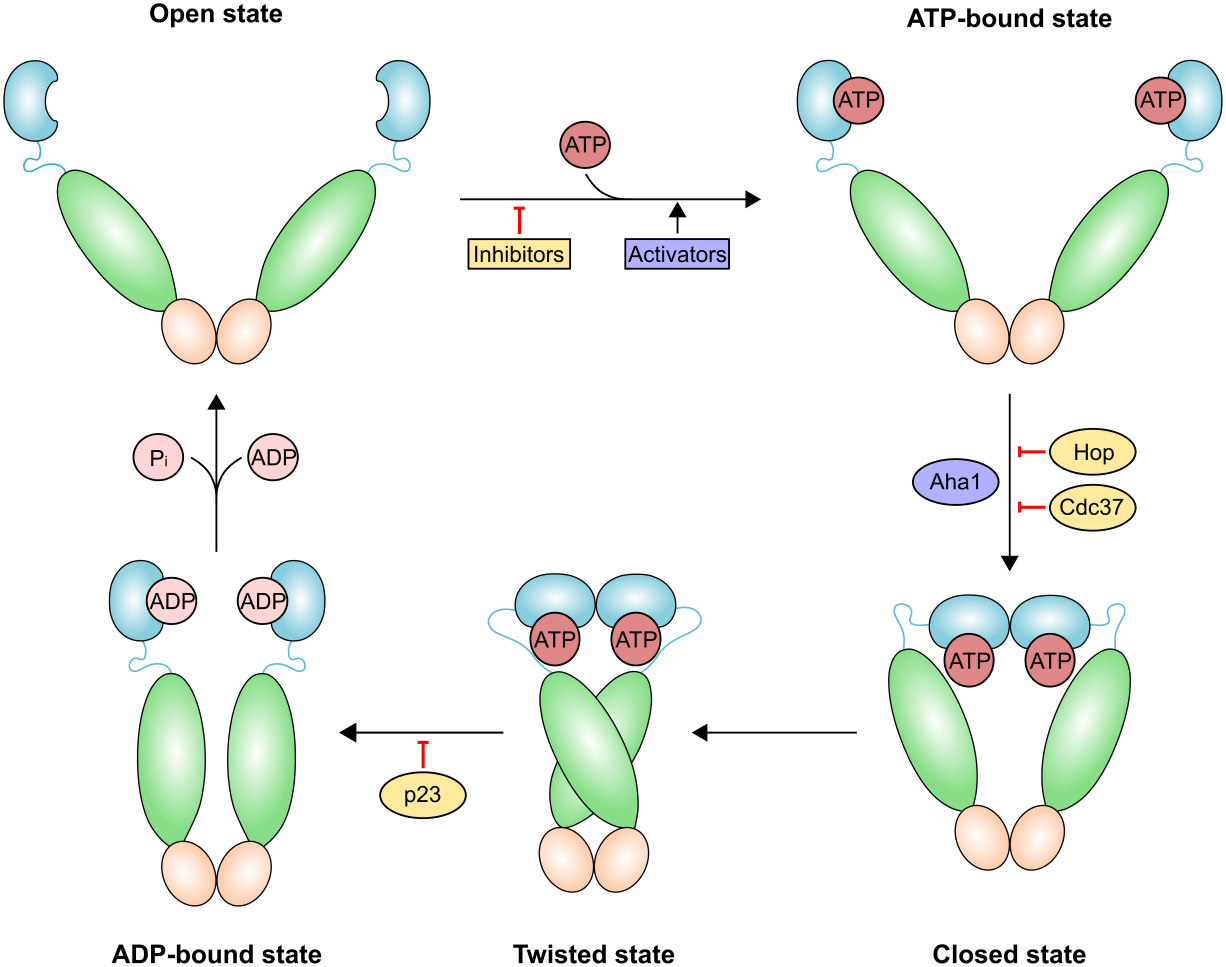
Schematic representation of the HSP90 conformational cycle. HSP90 domains are shown in different hues and colored blue [ATP-bound, ADP-bound or naked N-terminal domain (NTD)], green [middle domain (MD)] and orange [C-terminal domain (CTD)]. Binding of the co-chaperone activator of Hsp90 ATPase homolog 1 (AHA1) or allosteric HSP90 activators promote the formation of the closed state and increase the rate of ATP hydrolysis. The co-chaperones HOP (also known as STIP1) and cell division cycle 37 homolog (CDC37) and HSP90 inhibitors exert an opposite effect by preventing the structural changes necessary for the completion of the cycle. Prostaglandin E synthase 3 (p23; also known as PTGES3) slows the HSP90 ATPase cycle by stabilizing the twisted state.

Dimers of HSP90 family proteins undergo a complex functional cycle that might allow them to adapt to different client proteins. ATP binding elicits a series of conformational changes (Figure 2) leading to the “closed conformation” of the chaperone in which ATP hydrolysis occurs. Induction of the closed state is the rate-limiting step of the reaction. ATP binding has a much lower affinity than ADP binding (K_D_ ~400 μM vs. ~10 μM), indicating that under physiological conditions of nucleotide concentrations, cytosolic Hsp90 primarily populates two states that are absent in ATP-regenerating conditions: either ADP bound to both arms, or ATP bound to one arm and ADP bound to the opposite arm (30). A NTD loop termed the “lid” region closes over the ATP-bound active site. NTDs then dimerize and associate with the M-domains, prompting ATP hydrolysis (31). This step is instrumental for dissociation of the two NTDs and the subsequent release of ADP and inorganic phosphate (Pi); eventually, HSP90 returns to the open (*apo*) conformation.

A dynamic equilibrium exists among the different conformations of HSP90. X-ray crystallography, small-angle X-ray scattering (SAXS) solution data and kinetic measurements have led to the proposal of a general functional mechanism based on global conformational modulations triggered by ATP binding and hydrolysis, which integrates an array of structural information (25). In the absence of nucleotide, various conformations co-exist. ATP binding shifts the chaperone to a partially closed state, and then into a closed conformation; in the case of TRAP1, this is asymmetric and significantly strained, leading to buckling of the MD:CTD interface (32). The hydrolysis of ATP is sequential and deterministic and determines the conformational modulation of the MD:CTD region (32). This region has a key role in client binding (33) and is close to the allosteric C-terminal binding site. Upon ATP hydrolysis, the strain is relieved to yield a symmetric closed state (23). *In vitro* experiments demonstrate that although the fundamental conformational states are well-conserved among species and paralogs, equilibria and kinetics are unique for every HSP90 homolog (26), suggesting adaptations to the specific needs of clients in each subcellular environment.

In cells, HSP90 acts as a nucleating site for the assembly of networks of stable multiprotein complexes that show tumor-specific traits of physical and functional integration absent in normal cells (34, 35). Such large complexes act to enhance biochemical and metabolic pathways required to bear conditions encountered during malignant transformation. Mechanistically, co-chaperones select stochastically distributed HSP90 conformers that meet functional needs and structurally organize complexes for client activation (e.g., Cdc37 for kinases), or either increase (e.g., Aha1) or slow down (e.g., p23) ATPase rates of HSP90. Some co-chaperones, for instance Aha1 and Cdc37, are overexpressed in cancer and can be post-translationally modified by HSP90 client enzymes, generating reciprocal regulatory mechanisms of the chaperone machinery (36). By using the extraordinary power of state-of-the-art cryoEM, the Agard lab revealed the structures of two very different client HSP90 complexes, namely HSP90:Cdc37:Cdk4 (37) and HSP90:Hsp70:Hop:GR (38). Expectedly, the multiprotein functional assemblies are quite dynamic, which explains why they have eluded crystallization.

The rate of ATP hydrolysis by HSP90 is low, thus the HSP90 cycle may be differently tuned within different tissues or subcellular compartments by complex post-translational modifications (PTMs) that include phosphorylation, sumoylation, acetylation, S-nitrosylation, oxidation, and ubiquitination (20, 36, 39) (Table 1). We are far from understanding the effect of individual PTMs. In general, HSP90 phosphorylation, predominantly on Ser residues, but also on Thr and Tyr residues (68), slows down the chaperone conformational cycle, affecting maturation of clients and interactions with co-chaperones (20, 69). Co-chaperones broaden the functional range of HSP90, either modulating its chaperone cycle or enabling the recruitment of specific subsets of clients, thus providing a suitable folding platform for each client, or even carrying out both activities (12). Co-chaperone binding is also regulated by HSP90 acetylation, whereas S-nitrosylation in the CTD inhibit HSP90 chaperone cycle and activity (65, 68). Furthermore, PTMs can function as allosteric switch points that regulate interdomain communication between the two protomers (65, 69).

**Table 1:** Summary of the most relevant post-translational modifications of HSP90.

**Hsp90 residue**		**Modification**	**Enzyme**	**Main effect**	**References**
**α**	**β**				
T5, T7	N/A	Phosphorylation	DNA-PK	Unknown	(40)
T36	T31	Phosphorylation	CK2	Decreased ATPase activity, increased inhibitor sensitivity	(41)
Y38	Y33	Phosphorylation	Swe1/Wee1	Increased client association, decreased inhibitor binding	(42)
Y61, Y284, Y492, Y604	Y56, Y276, Y484, Y596	Phosphorylation	Unknown	Unknown	(43–45)
S63, T65	S58, T60	Phosphorylation	CK2	Unknown	(46)
K69, K100, K292, K327, K478, K546, K558	K64, K284, K319	Acetylation	HDAC6, p300	Increased inhibitor binding, increased extracellular location and association with MMP-2	(47)
T88	T83	Phosphorylation	PKA	Increased translocation	(48)
T90	T85	Phosphorylation	PKA	Increased association with some co-chaperones and client proteins	(49)
K112, K283, K292, K407, K546, K558, K615, K631	K107, K275, K284, K399, K538, K550, K607, K623	Ubiquitination	CHIP	Inactivation and degradation of HSP90	(50, 51)
T115, T425, T603	N/A	Phosphorylation	PKCγ, Mps1	Decreased chaperone activity, increased inhibitor sensitivity	(52, 53)
K191	N/A	Sumoylation	Unknown	Increased association with Aha1, increased inhibitors binding	(54)
Y197	Y192	Phosphorylation	Yes kinase	Decreased association with Cdc37	(55)
S231, S263	S226, S255	Phosphorylation	CK2	Increased apoptosome formation, decreased client association	(56, 57)
K294	K286	Acetylation	HDCA6	Decreased affinity for HSP70, co-chaperones, and client proteins	(58)
Y309	Y301	Phosphorylation	c-Src	Increased association with eNOS	(59)
Y313	Y305	Phosphorylation	Yes kinase	Increased association with Aha1	(55)
S391, T624	T89, S383, T616	Phosphorylation	Pnck, PKA	Dissociation of client proteins	(60, 61)
S460	S452	Phosphorylation	PKA	Unknown	(62)
C572	N/A	Oxidation	Unknown	Decreased chaperoning activity	(63)
S595	S587	Phosphorylation	p38γ	Formation of a ternary complex with mutated K-Ras and p38γ	(64)
C597	C589	S-nitrosylation	eNOS	Decreased ATPase activity	(65, 66)
Y627	Y619	Phosphorylation	Yes kinase	Dissociation of clients and co-chaperones	(55)
T725, S726	S718	Phosphorylation	CK1, CK2, GSK3-β	Decreased CHIP binding, increased HOP binding	(67)

*Aha1, HSP90 ATPase homolog 1; Cdc37, cell division cycle 37 homolog; CK1, casein kinase 1; CK2, casein kinase 2; CHIP, C-terminus of Hsp70-interacting protein; DNA-PK, DNA-dependent protein kinase; eNOS, endothelial nitric oxide synthase; GSK3-β, glycogen synthase kinase beta 3; HDAC6, histone deacetylase 6; MMP-2, matrix metalloproteinase-2; Mps1, monopolar spindle-1; PKA, protein kinase A; PKCγ, protein kinase C gamma; Pnck, pregnancy-upregulated non-ubiquitous calmodulin kinase; PP5, serine/threonine-protein phosphatase 5*.

Despite this level of depth and sophistication in the knowledge of the roles of various players in the chaperone cycle, the factors that determine whether a protein is a HSP90 client are still elusive. HSP90 might facilitate conformational rearrangements in clients, or it might sequester them, thus avoiding their proteasomal degradation (70, 71).

Furthermore, protein quality control is a compartmentalized process characterized by peculiar features in the various subcellular regions, where different and specific networks of chaperones are present. In mitochondria, which house essential metabolic pathways, such as the tricarboxylic acid (TCA) cycle, the oxidative phosphorylation (OXPHOS) machinery and branches of amino acid, lipid, and nucleotide metabolic pathways, the paralog of the HSP90 chaperone family is TRAP1 (15, 72). TRAP1 shares the same domain structure of HSP90, but lacks a charged linker between middle and C-terminal domains and displays a long N-terminal extension called “strap” that extends between protomers in the closed state and inhibits its function at low temperatures. During TRAP1 chaperone cycle, ATP binding induces a dramatic structural change from the *apo*, open state to a closed, asymmetric structure, with one protomer buckled and the other one in a straight conformation (25, 73–75). The subsequent hydrolysis of the two ATP molecules bound to the TRAP1 dimer gives off the energy required for client remodeling. Hydrolysis of the first ATP swaps protomer symmetry and rearranges the client-binding site, causing structural changes in client conformation, whereas the second ATP is used to induce the formation of a compact ADP state of the chaperone, which releases the client and eventually the ADP molecules (32). Interestingly, work by the Agard lab has established that the asymmetric theme in the mechanisms of conformational dynamics is a general characteristic of the Hsp90 family (25, 32, 74). We are just beginning to understand PTM regulation of TRAP1 (Table 2), whereas no co-chaperones are known and the number of its known clients remains quite small.

**Table 2:** Reported post-translational modifications of TRAP1.

**TRAP1 residue**	**Modification**	**Enzyme**	**Main effect**	**References**
S501	S-nitrosylation	NOSs	Decreased ATPase activity, enhanced proteasomal degradation of TRAP1	(76, 77)
S511, S568	Phosphorylation	ERK1/2	Formation of a multimeric complex with SDH and ERK1/2, increased SDH inhibition and neoplastic growth	(78)
Unknown	Phosphorylation	PINK1	Prevent oxidative stress-induced apoptosis	(79)
Unknown	Deacetylation	SIRT3	Increased mitochondrial respiration under low glucose conditions	(80)
Unknown	Phosphorylation	c-Src	Inhibition of complex IV activity	(81)

*ERK1/2, extracellular signal-regulated kinases 1 and 2; NOSs, nitric oxide synthases; PINK1, PTEN-induced kinase 1; SIRT3, sirtuin 3*.

## HSP90 Chaperones in Cancer

Tumor cells are exposed to a variety of stresses that can directly hit polypeptide conformation and functionality, including unbalance in redox equilibrium caused by a profound rewiring of their metabolic circuitries and by inconstant oxygen availability (82), thus leading to a potential increase in oxidative stress. Moreover, genomic instability in a framework of relentless proliferation can lead to a high risk of synthesis of misfolded proteins. HSP90 molecular chaperones are central hubs of complex biological pathways and strongly induced by hypoxia, shortage of nutrients, high rate of DNA replication and expression of mutated proteins, thus acting at several levels to block cell death and to promote proliferation under the harsh conditions of neoplastic progression (22).

Indeed, overexpression of HSP90 has been observed in a variety of cancer types, including medulloblastoma, pancreatic, ovarian, breast, lung, and endometrial cancer, oropharyngeal squamous cell carcinoma and multiple myeloma, and high HSP90 levels are associated with poor prognosis in lung, esophageal and bladder cancer, melanoma and in several forms of leukemia (13). Most identified HSP90 clients are proteins related to biological processes dysregulated in cancer, such as signal transduction, survival, growth and invasiveness of cells and include steroid hormone receptors, both wild-type and mutant forms of the tumor suppressor p53, telomerase, hypoxia-inducible factor 1α (HIF1α) (12) and kinases, which display a continuous range of binding affinities for HSP90 (83). Some kinases would require HSP90 to stabilize their open conformation in order to efficiently bind ATP, whilst others seem to only need HSP90 for initial folding (27, 84) and would perform their enzymatic activity without HSP90 assistance. Studies with closely related pairs of client/non-client kinases, like the client v-Src and the non-client c-Src, which share 98% sequence identity, suggest that HSP90 dependence requires a combination of factors, including folding cooperativity and subtle changes in the overall stability and compactness of clients (85). HSP90 can also be secreted in a variety of tumor cells under the regulation of HIF-1α. In the extracellular matrix and on cell surfaces, HSP90 decreases the tumor-suppressing effects of TGFβ and modulates cell migration and invasiveness (17), for instance by interaction with matrix metalloproteases (86–88).

Further layers of complexity exist in the interplay between HSP90 chaperones and cancer. Some HSP90 clients, like p53, may change the chaperone and co-chaperone networks by inducing the expression of co-chaperone subsets (89). The “epichaperome” is a functionally connected network of HSP70 and HSP90 machineries, which includes co-chaperones and is present in more than 50% of tumors (35), where it expands and integrates chaperone activities and promotes tumor survival (12).

**Figure 3:**
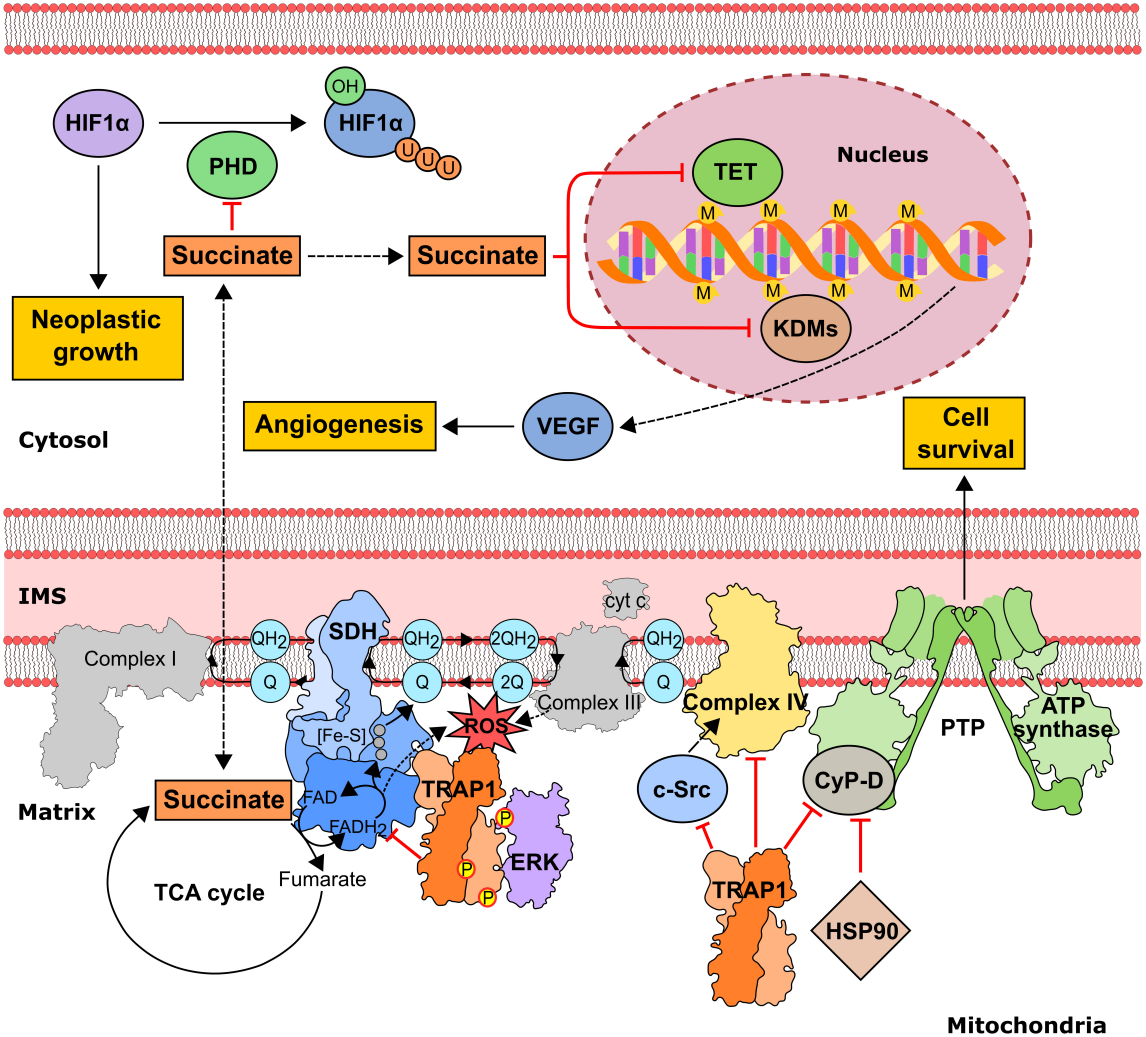
Pro-neoplastic effects of the mitochondrial chaperone TRAP1 in tumor cells. TRAP1 downregulates OXPHOS by inhibiting both succinate dehydrogenase (SDH) and cytochrome *c* oxidase (*aka* complex II and complex IV of the mitochondrial respiratory chain, respectively). SDH down-regulation is enhanced by ERK1/2-dependent phosphorylation of TRAP1 and leads to an increase in intracellular succinate concentration. In turn, succinate inhibits the dioxygenase families PHD (prolyl hydroxylase), preventing proteasomal degradation of the pro-neoplastic transcription factor HIF1α, TET (10–11 translocation family of methylcytosine hydroxylases) and KDMs (histone lysine demethylases), thus prompting alterations in gene expression through epigenetic modifications. TRAP1-dependent down-modulation of Complex IV activity involves inhibition of mitochondrial c-Src. TRAP1 also protects from cell death by inhibiting the opening of the permeability transition pore (PTP) and interacts with the PTP regulator cyclophilin D (CyP-D). IMS, intermembrane space; M, methylation; P, phosphorylation; U, ubiquitination.

Cancer cells incur high level of mitochondrial functional changes in order to maintain the required levels of ATP, reducing equivalents and metabolic intermediates, and exposure to fluctuating levels of oxygen and to high amounts of ROS can hamper proper protein folding and lead to mtDNA mutations, thus damaging mitochondrial structure and function (10, 90). Under these conditions TRAP1, the mitochondrial HSP90 paralog, could contribute to maintain an adequate quality control and to preserve mitochondrial functions. TRAP1 expression is higher in many tumors compared to surrounding non-malignant tissues and was shown to correlate with progression, metastasis and disease recurrence in prostate and breast cancer, hepatocellular and colorectal carcinoma and non-small cell lung cancer (15, 91). In mitochondria, TRAP1 provides resistance to oxidative stress (18, 92), possibly counteracting the effects of several chemotherapeutics, and inhibits opening of the permeability transition pore (PTP), a cell death-inducing mitochondrial channel composed by the ATP synthase holoenzyme and that can be induced by a ROS surge (93). Thus, TRAP1 exerts a pro-neoplastic function by counteracting ROS-induced, PTP-mediated cell death. However, ROS effects on tumor growth are multifaceted, as oxidative stress can favor genetic instability and aggressiveness of tumors in advanced stages. Consequently, the anti-oxidant activity of TRAP1 could hamper growth in specific tumor types or stages as in cervical carcinoma, clear cell renal cell carcinoma and high-grade ovarian cancer, where TRAP1 expression inversely correlates with tumor grade (81, 94). TRAP1 also down-regulates the activity of both cytochrome *c* oxidase, the complex IV of the respiratory chain (81), and of succinate dehydrogenase (SDH) (95), which oxidizes succinate to fumarate at the crossroad between OXPHOS and the TCA cycle (Figure 3). Hence, TRAP1 participates in the metabolic switch of tumor cells toward aerobic glycolysis, i.e., decreased OXPHOS activity paralleled by enhanced glucose utilization (96). Importantly, SDH inhibition increases intracellular succinate levels, and succinate acts as an oncometabolite in several ways (97). It competitively inhibits α-ketoglutarate–dependent dioxygenases that include prolyl hydroxylases (PHDs), the JmjC domain-containing demethylases (KDMs) and the TET (10–11 translocation) family of 5-methylcytosine hydroxylases (7). PHD inhibition stabilizes the transcription factor HIF1α, increasing invasiveness, angiogenesis and further metabolic changes in tumor cells (98), whereas inhibition of KDMs, which hydroxylate lysine residue on histones, and of TETs, which induce DNA demethylation of CpG islands near gene promoters, prompts complex epigenetic rearrangements in neoplastic cells (Figure 3). Oncogenic kinase pathways directly target TRAP1, as it is both Tyr-phosphorylated in a Src-dependent way and Ser-phosphorylated by ERK1/2, favoring cytochrome oxidase inhibition and enhancing TRAP1 inhibition of SDH activity, respectively (78, 81), whereas S-nitrosylation elicits TRAP1 degradation (76) and decreases its ATPase activity (77) (Table 2).

## Inhibiting HSP90 As an Anti-Neoplastic Strategy

To a superficial analysis, HSP90 would appear an unlikely target for anti-neoplastic drugs, as it is highly expressed in all cell types. However, HSP90 inhibitors tend to accumulate in tumors and are more toxic in most cancer cells than in their non-transformed counterparts (99). This could depend on HSP90 induction and/or ectopic localization in many tumor types, where it can undergo selective PTMs and can interact with a specific landscape of co-chaperones and client proteins, creating multimolecular complexes restricted to tumor cells. For instance, HSP90 binds inhibitors more strongly when it is part of epichaperome complexes (35). Some HSP90 clients express oncogenic mutations that can change their association pattern with HSP90. This can create interactions that are selectively druggable and enhance HSP90 affinity for inhibitors (100). Moreover, oncoproteins could become addicted to HSP90 in order to maintain their mutated and potentially unstable conformations, rendering disruption of this interaction particularly toxic for neoplastic cells (101).

To date, more than 50 clinical trials have been performed or are under way with several HSP90 inhibitors, but expectations of evolution toward therapeutic application have been largely frustrated. In most cases, the anti-neoplastic efficacy of HSP90 inhibition has been modest, only inducing a transient growth arrest that is reverted after drug removal, or adverse effects have been recorded, leading to termination or suspension of clinical trials. Possible reasons of these failures include a compensatory induction in the expression of other HSPs, in particular HSP70, off-target effects in patients caused by the multiplicity of biological functions regulated by HSP90 and insufficient stratification of patients enrolled in the studies (12).

### ATP-Competitive Inhibitors

Since HSP90 functions depend on its ATPase activity, most drugs have been developed as competitive inhibitors targeting the active site and competing with ATP for binding the protein. The underlying hypothesis is that disrupting the enzymatic activity of HSP90 would reverberate on the chaperone protein folding machinery, simultaneously affecting multiple oncoproteins that are essential to the proliferation and maintenance of cancer cells. The molecular basis is that ATP-ADP exchange regulates and determines a well-balanced, functionally-oriented conformational equilibrium: outcompeting the nucleotide by drug-like ligands will expectedly unbalance conformational dynamics, leading to a blockage of correct biological activities.

The unusual mode of ATP binding to HSP90 allows specific inhibition of HSP90 chaperone activity by chemical compounds. Indeed, the base and the sugar of ATP are lodged in the NTD binding pocket in a “kinked” conformation, with the phosphates pointing outwards and the γ-phosphate becoming buried only when MD and NTD associate (102). Competitive inhibitors in the ATP pocket block ATP hydrolysis and subsequently hamper the closure of the N-terminus of the dimer, thus inhibiting the HSP90 chaperone cycle (103) (Table 3). Prototypical molecules of this class are the benzoquinone geldanamycin (GA) and the macrolide radicicol, whose selectivity has been widely discussed elsewhere (121, 122). Both compounds demonstrate strong toxicity on tumor cells (20). GA was the first HSP90 inhibitor to be evaluated as an antitumor agent. In neoplastic cells GA induces apoptosis, inhibits cell migration associated with FAK and HGF activity, as well as angiogenesis and epithelial-mesenchymal transition by down-regulating VEGF receptor, HIF-1α and NF-κB signaling (123–126). However, GA preclinical trials were discontinued due to its hepatotoxicity, poor solubility and *in vivo* instability. Similarly, radicicol cannot be used as a drug as it is not stable (13, 19). Therefore, several radicicol and GA derivatives were developed (127, 128). Tanespimycin (17-N-allylamino-17-demethoxygeldanamycin, 17-AAG) is a GA derivative that induces cell cycle arrest and apoptosis in cancer cells. Tanespimycin entered a Phase III clinical trial for multiple myeloma, but its development was halted because of poor solubility and poor oral bioavailability and lapsed patent protection, with a prolonged disease stabilization in several tumor types, without any tumor regression (13). Another GA derivative, Alvespimycin (dimethylaminoethylamino-17-demethoxygeldanamycin, 17-DMAG) demonstrated anti-tumor activity and improved solubility in water, but dose-limiting side effects were recorder during various clinical trials (129, 130) (around 2000 X ray structures can be found at the RCSB protein databank (https://www.rcsb.org/). A new generation of GA derivatives is currently under evaluation, such as retaspimycin hydrochloride (IPI-504) or ganetespib. These molecules are well-tolerated and effective in cells, with reduced liver and cardiovascular toxicity (131, 132). Ganetespib (STA-9090), a small molecule inhibitor containing a triazole moiety that binds to the ATP-binding pocket of HSP90, is the most promising second generation HSP90-targeting compound (133). Its potent anti-tumor activity was translated into several clinical studies, demonstrating efficacy both in monotherapy and in combination with other drugs in various cancer types. Ganetespib produced significant single agent activity in ALK-driven cancer models, however only transient responses were reported in patients with KRAS mutant tumors due to rapid development of resistance (134). Second generation radicicol derivatives, such as NVP-AUY922 (luminespib, VER-2296) or AT13387 (Onalespib) showed strong efficacy both pre-clinically and in clinical trials, some of which are ongoing (103). Other inhibitors targeting the HSP90 ATP-binding pocket include purine analogs that can be administered orally (135), substituted resorcinols and compounds featuring a substituted benzamide substructure. Many of these molecules have entered clinical trials (136).

**Table 3:** Key data concerning orthosteric Hsp90 inhibitors Geldanamycin, Radicicol, and PU-H71.

**Inhibitor**	**Structure**	**Selected bioactivity indicators^*a*^**	**Contact residues^*b*^**	**PDB^*c*^**	**Clinical trials^*d*^**	**Other targets^*e*^**
Geldana mycin	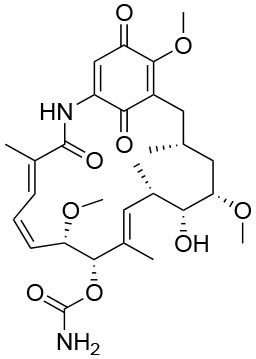	IC_50_^*f*^ = 15.8 nM (104) EC_50_^*g*^ = 30 nM (105) K_d_^*h*^ = 1.35 μM (106)	N51, S52, D54, A55, K58, D93, I96, M98, D102, N106, L107, K112, G135, V136, G137, F138, Y139	1YET (Hsp90) (107)	(None active or recruiting)	Nitric oxide synthase (104); oncogene product p185^erbB−2^ protein tyrosine-kinase (104, 108, 109); src protein tyrosine-kinase (110)
Radicicol	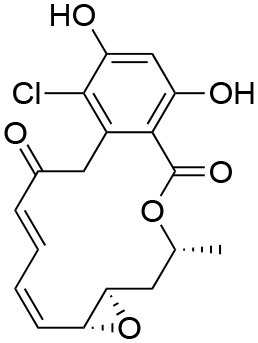	IC_50_^*f*^ = 37.5 nM (104) EC_50_^*i*^ = 25 nM (111) K_d_^*j*^ = 2.2 nM (112), 3.04 nM (113)	L48, N51, S52, D54, A55, K58, D93, I96, M98, N106, L107, F138, T184, V186	4EGK (Hsp90) (114)	(None active or recruiting)	Topoisomerase VI (115); Pyruvate dehydrogenase kinase (116); Carbonyl reductase 1 (117);
PU-H71	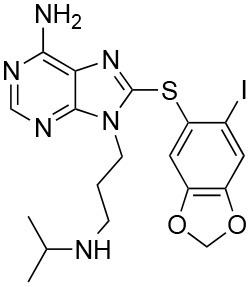	IC_50_^*f*^ = 50 nM (118) EC_50_^*k*^ = 16 nM (118) K_d_^*h*^ = 780 nM (106)	N51, S52, A55, K58, D93, I96, G97, M98, L107, F138, Y139, V150, W162, T184	2FWZ (Hsp90) (119)4Z1F (Trap1) (120)	**Phase I:** • metastatic breast cancer (with nab-paclitaxel) • safety and tolerability (with ruxolitinib) • advanced malignancies	(None known)

a *Chosen bioassays are limited to those that are directly comparable or similar in nature. Only human chaperones and cell lines have been considered. The list is merely indicative: a more complete list is available from the PubChem Database (https://pubchem.ncbi.nlm.nih.gov/compound/5288382; https://pubchem.ncbi.nlm.nih.gov/compound/radicicol; https://pubchem.ncbi.nlm.nih.gov/compound/pu-h71 [accessed May 24, 2020])*.

b *Residues with at least one atom detected to be closer than 3.0 Å to any atom in the ligand. Hydrogen atoms included in the analysis*.

c *Human chaperone targets only*.

d *Active and/or recruiting clinical trials only, as listed in the PubChem Database (https://pubchem.ncbi.nlm.nih.gov/compound/5288382; https://pubchem.ncbi.nlm.nih.gov/compound/radicicol; https://pubchem.ncbi.nlm.nih.gov/compound/pu-h71 [accessed May 24, 2020])*.

e *Documented, non-chaperone human targets only*.

f *Antiproliferation/growth inhibition of SkBr3 breast cancer cell line*.

g *Antiproliferative activity against HCT116 cells (luminescence assay)*.

h *Hsp90 competition with fluorescent geldanamycin (fluorescence assay)*.

i *Hsp90 binding (Western Blot on MCF-7 cell lysate)*.

j *Hsp90 binding (surface plasmon resonance analysis)*.

k *Hsp90 competition with fluorescent geldanamycin (fluorescence assay on SkBr3 cell lysate)*.

The nucleotide-binding pocket of the NTD is considered the most conserved structural component among HSP90 family members, impeding the rational design of paralog-selective inhibitors targeting it. Therefore, a strategy used for inhibiting TRAP1 was to exploit its subcellular localization, and mitochondria-permeable GA derivatives were conceived and synthesized. Gamitrinibs (Geldanamycin mitochondrial matrix inhibitors) are 17-AAG derivatives linked to either guanidinium repeats or triphenylphosphonium (TPP), used as mitochondriotropic moieties (137). These molecules induced mitochondrial PTP opening and tumor cell death in mouse models of prostate cancer (138). Similarly, TPP was linked to the purine-scaffold Hsp90 inhibitor PU-H71PU-H71 (120) to develop a mitochondria-targeting conjugate, SMTIN-P01. Indeed, the co-crystal structures of PU-H71 in complex with either HSP90 or TRAP1 highlighted slight differences in the ATP-binding pocket of the two chaperones. The Leu172-Phe201 sequence is disordered only in the TRAP1 binding site, and the two flanking residues (Asn171 and Gly202) have different configurations (103, 139), providing a molecular basis for the differentiation of ligands to target specifically one isoform. Accordingly, TRAP1 inhibitors without mitochondrial delivery vehicles were also reported, showing a better binding to TRAP1 than to Hsp90 (139).

In spite of this wealth of efforts, so far ATP-competitive HSP90 inhibitors have not met clinical expectations and none of them has been approved for cancer treatment. All compounds showed toxicity (e.g., liver or ocular toxicity) and/or absence of convincing anticancer efficacy (13). Possible reasons include only partial inhibition of the target client proteins, P-glycoprotein-dependent efflux from target cells, requirement for reductive metabolism to reach full activation (17-AAG), off-target effects on biochemical pathways not specific of tumor cells (2). Moreover, the drug concentration required to outcompete ATP and induce client degradation is often the same as that needed to induce the heat shock response (HSR). HSR is based on the activation of the transcription factor HSF1, which leads to overexpression of multiple heat shock proteins, including HSP70, HSP40, and HSP27. As HSR is a survival mechanism, it can be detrimental in an anti-cancer therapy, and trying to avoid it determines dosage, toxicity and tolerance problems (136).

These limitations could be overcome if one were able to disentangle the intricacies in the functional mechanisms of different paralogs together with their relationships to metabolic and/or signaling regulation. Chemical interventions based on using *ad hoc* designed molecules to perturb a specific aspect of the HSP90 functional spectrum and directly report on the consequences of this perturbation would represent ideal tools. The diversity of conformations, protein-protein interactions, and functions involved in HSP90 mechanisms makes the “one-drug-fits-all” perspective unrealistic. On the other hand, that very diversity may provide a greater number of drug discovery opportunities thanks to the variety of structural and chemical motifs involved in conformational regulation and protein-interaction phenomena.

### Allosteric Inhibitors

The perspective of developing different strategies to target various aspects of the multifaceted HSP90 complexes can expectedly generate novel types of chemical intervention, unveiling new chemical tools for the investigation of biological mechanisms and/or novel candidates for therapeutic applications. In this conceptual framework, increasing appreciation is being given to the potential of allosteric modulators (Table 4). Allostery defines the feature of proteins to undergo a modulation of affinity toward a primary binding event caused by binding an “effector” a different distant position called the allosteric site. This modulation may cause increase or decrease of protein activity (ATP in Hsp90 processing for instance) and its downstream effects in the cell. Allosteric modulators represent an interesting opportunity for drug development in HSP90-related metabolic circuitries for several reasons: on the one hand, they permit to finely tune and regulate both the enzymatic functions and the interactions of HSP90, and on the other hand they may facilitate the selective targeting of different chaperone isoforms. The latter option would be highly desirable in the development of drugs that need to perturb the function of one specific paralog of the protein, active in specific pathologic conditions and/or in specific subcellular compartments. This type of chemical tools could selectively regulate/disrupt the functions of paralogs in a controlled way, shedding light on the correlations between the induced perturbation and the consequent biological activities, and laying the bases for novel mechanism-driven therapeutic interventions. Allostery is the prime mechanism by which achieving fine protein regulation via the activation of specific conformational states that meet functional requirements. Modifications at one site, caused for instance by ligand binding, are propagated through the protein, shifting the structural population with the modulation/perturbation of dynamic states that encode specific functions. In this context, the atomistic understanding of allosteric mechanisms provides the basis for the development of new drug candidates. In HSP90, the region at the border between the M-Domain and the C-terminal domain, located at 60Å from the ATP-site, has been shown to host a druggable allosteric site (152–156).

**Table 4:** Summary of the most important compounds identified as allosteric modulators of HSP90.

**Name**	**Structure**	**Pharmacokinetics**	**Mechanism**	**Residues**	**References**
Novo biocin	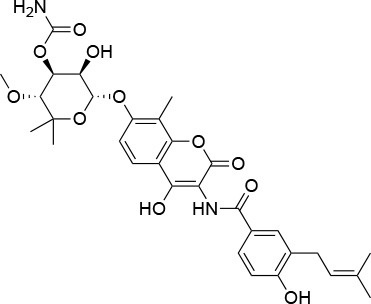	IC_50_ (SkBr3) _~_700 μM^*a*^	Disruption of the interaction with the co-chaperones Hsc70 and p23	L663—H676	(140–142)
Chloro biocin	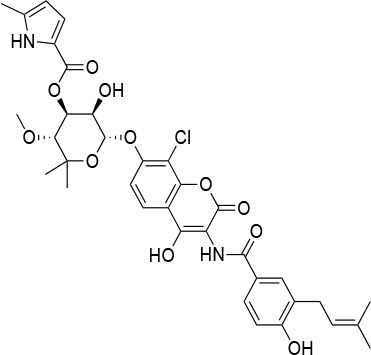	IC_50_ (SkBr3) _~_60 μM^*a*^	Unknown	Unknown	(141, 142)
**15a**	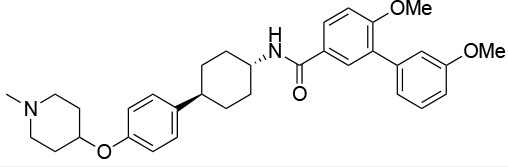	IC_50_ (SkBr3) _~_0.17 ± 0.02 μM^*b*^ IC_50_ (MCF-7) _~_0.22 ± 0.01 μM^*b*^	Unknown	Unknown	(143)
**80c**	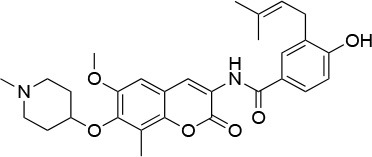	IC_50_ (SkBr3) _~_0.42 ± 0.01 μM^*b*^ IC_50_ (MCF-7) _~_0.54 ± 0.02 μM^*b*^	Unknown	Unknown	(144)
Derrubone	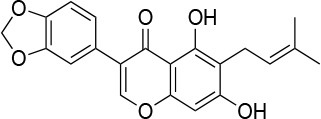	IC_50__~_ 0.23 ± 0.04 μM^*c*^ IC_50_ (SkBr3) _~_12 ± 0.3 μM^*b*^ IC_50_ (MCF-7) _~_9 ± 0.7 μM^*b*^	Stabilization of HSP90-client interactions	Unknown	(145)
Withaferin A	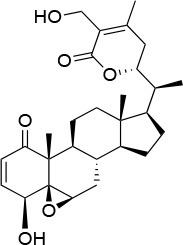	IC_50_ (Panc-1) _~_1.24 μM^*b*^ IC_50_ (MiaPaCa2) _~_2.78 μM^*b*^	Disruption of the HSP90-Cdc37 complex in an ATP-independent way	Unknown	(146)
Celastrol	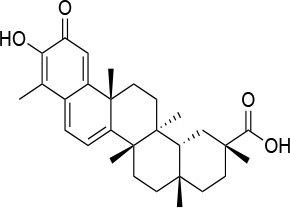	IC_50_ (Panc-1) _~_3 μM^*b*^ IC_50_ (Hep3B) _~_0.3 ± 0.08 μM^*b*^	Disruption of the association between HSP90 and Cdc37	T94 – M125	(147, 148)
**19**	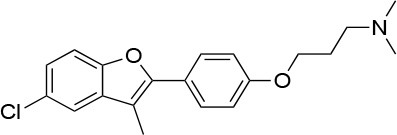	IC_50_ (DU145) _~_12.7 ± 2.5 μM^*b*^ IC_50_ (STO) _~_9.1 ± 1.1 μM^*b*^	Acceleration of the HSP90 conformational cycle	E477, D503 (protomer A), R591 (protomer B)	(149)
**25**	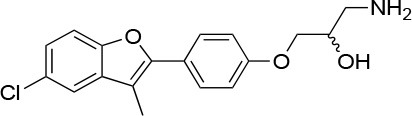	IC_50_ (STO) _~_22.1 ± 1.1 μM^*b*^	Increased HSP90 ATPase activity favoring its active state	E477, R591 (protomer A), K594 (protomer B)	(150)
LA1011	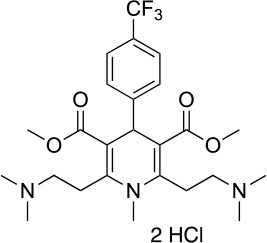	K_d_ (HSP90α) _~_3.8 ± 0.7 μM^*d*^ K_d_ (HSP90β) _~_9.7 ± 0.7 μM^*d*^	Alteration of HSP90 chaperoning activity	G675, S677, L678	(151)

a *Expression levels of HSP90 client proteins*.

b *Anti-proliferation assay*.

c *Luciferase refolding*.

d *Isothermal titration calorimetry*.

Experimental evidence for the possibility to target this site initially came from the Neckers group, who demonstrated the interaction between HSP90 and coumarin (140, 141). In particular, they observed that the coumarin antibiotics Novobiocin and Chlorobiocin caused the impairment of HSP90 chaperone functions by disrupting the interactions with the large group of TPR-containing co-chaperones. Importantly, despite being used at high concentrations, these molecules did not show the toxicity of ATP-competitive ligands related to the induction of the HSR (12). Novobiocin causes moderate anti-proliferative effects on tumor cell models, down-regulating the expression of important HSP90-dependent clients, including Raf-1, erbB2, mutant p53, and v-Src (141). Since this seminal demonstration of the importance and druggability of alternative binding sites, significant synthetic efforts have been dedicated to improving the activities of coumarin-based allosteric molecules. In this context, the Blagg group demonstrated the possibility of substituting the carbohydrate moiety and the phenyl substituents around the coumarin scaffold by more accessible groups. The coumarin was also substituted by a series of biphenyl- or bi-cyclic scaffolds that permitted to explore the structure-activity relationships of a large number of derivatives. This series of allosteric inhibitors showed anticancer activities reaching the mid/low nanomolar range (143, 144). However, the anti-neoplastic efficacy of this family of inhibitors and their molecular mechanisms of action remain unclear (103), and no carboxy-terminal inhibitor has reached clinical trials up to now (85).

We have recently used a molecular dynamics-based strategy to identify an allosteric pocket distal to the ATPase site of TRAP1, which allowed the rational design and testing of small molecule compounds that target it (157). We have also found that the same allosteric domain can host the bis-dichloroacetate ester of the vegetal derivative honokiol DCA (HDCA) (158). All these molecules inhibit TRAP1 with a high selectivity over HSP90, abolishing TRAP1-dependent down-regulation of SDH activity in cancer cells and their *in vitro* tumorigenic growth (157, 158). Therefore, they constitute potential leads that can be used to better dissect TRAP1 biochemical functions and to conceive novel anti-neoplastic approaches.

Targeting Protein-Protein Interactions (PPIs) as sources of new leads is another interesting strategy to hit HSP90 chaperone function. PPIs are often less well-conserved than active sites, making easier to achieve selectivity (159). In general, PPIs tend to modulate the activity of the interacting proteins, rather than inducing on/off effects. Thus, PPI targeting compounds could flexibly titrate chaperone activity in the context of specific co-chaperone-client complexes, lowering the possibility of inducing off-target effects. However, not all PPIs are equally druggable, as the surfaces of contact usually have a larger buried surface area than enzyme-active sites, making it difficult to identify small molecules capable to block them (160).

Furthermore, as the pharmacological inhibition of clients or the downregulation of co-chaperone levels was shown to hypersensitize cells to HSP90 inhibitors, the perspective of combining drugs acting on different levels of regulation machineries is gaining increasing traction. Several compounds have been described that lead to the modulation of co-chaperone binding to HSP90. Examples include molecules like derrubone, withaferin A, and celastrol, which block CDC37 binding to HSP90. However, none of these compounds entered therapeutics so far because of insufficient efficacy in clinical studies. This could depend on several factors: variability and flexibility of HSP90 interactome in different cancer types and stages, off-target effects and HSR induction (161). Alternative interventions may involve direct targeting of PPI interfaces, disrupting HSP90-cochaperone interfaces, as recently reported (162).

### Allosteric Activators

**Figure 4:**
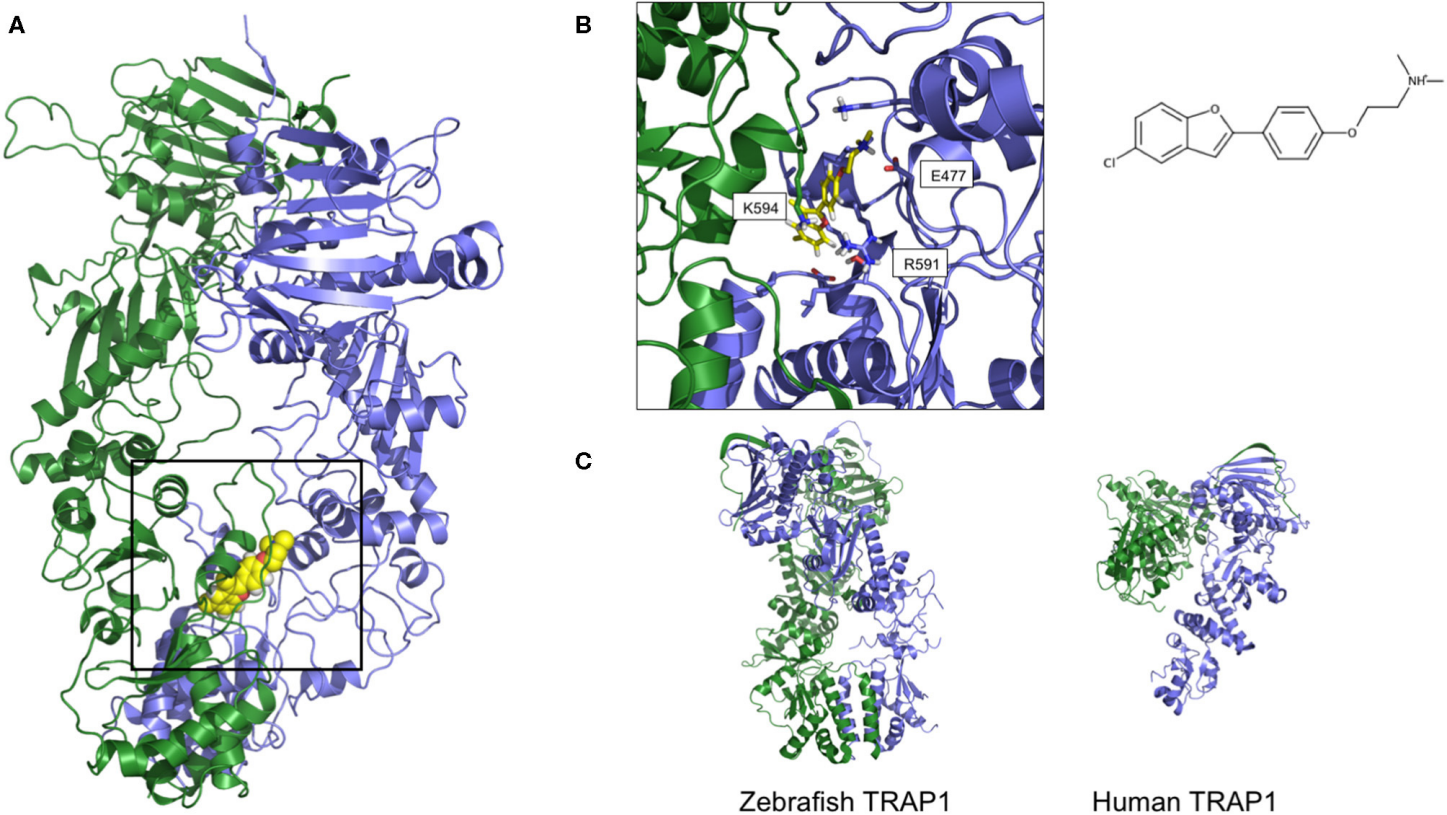
Two orientations of the predicted 3D structure of HSP90 with one allosteric activator bound. **(A)** The full-length structure of the complex, with the allosteric activator displayed as yellow van der Waals spheres. **(B)** Zoom on the structure of the activator and the contacting Hsp90 residues, together with its structure formula. The compound and the structure derive from simulations described in Sattin et al. (149), run starting from structure 2cg9.pdb **(C)** The 3D structures of Zebrafish and Human TRAP1, respectively from 6d14.pdb and 4z1i.pdb.

Together with inhibition, a viable approach to controlling the metabolic implications of HSP90 and its paralogs entails the use of allosteric activators of the ATPase and of the conformational dynamics of the chaperone. If we consider the dynamic nature of the HSP90 chaperone network, whereby different HSP90 conformations are stabilized by interactions with different multi-protein complexes, allosteric activators can expectedly select/favor a subset of HSP90 structures that may have preferential binding to a selected population of interactors (Figure 4). Starting from original methods of molecular dynamics (MD) simulation analysis, we were able to design a series of activators capable of modifying the biochemical properties of the chaperone, as well as its activities in cells (149, 150, 163). Conjugation to mitochondrial targeting moieties showed that designed activators could show activity also on TRAP1, modifying the activities of the client SDH, with an impact on the energy metabolism of targeted cells (150). In the context of activators, Prodromou and coworkers discovered dihydropyridines able to stimulate ATPase activity. Stimulation was shown to reverberate in a compromised ability to chaperone, which consequently induced the HSR in Alzheimer's disease cellular models (151).

The validity of the allosteric approach in studying the metabolic implications of chaperone stems from the fact that the overall activity of HSP90 is not simply shutdown but rather modulated. Ligand binding at a site far from the active site can be propagated through the protein, modifying the dynamic and/or structural populations with the modulation of dynamic states that encode specific interactions. This can in turn translate in the reshaping of surfaces that control interactions with other proteins (co-chaperones and clients), potentially favoring the binding of one partner over other alternatives. In this context, the controlled perturbation of interactions determines an overall modification of the dynamics in the protein networks that underlie signaling and metabolic pathways, providing novel intervention opportunities. Allosteric modulation of the chaperone enzymatic and conformational activities is thus directly coupled to phenotypic effects, through the finely-tuned modification of networks of PPIs.

## Coupling HSP90 Targeting with Other Anti-Neoplastic Approaches

The lack of translation to the clinical practice of HSP90 inhibitors clearly indicates the need of novel strategies to exploit cancer cell sensitivity to chaperone targeting. A promising treatment strategy is the combination therapy, in which HSP90-directed molecules are associated with other chemotherapeutics, and the combined action on different targets potently and selectively elicits the death of malignant cells (Table 5). Some examples already exist. A large scale phase III clinical trial (Galaxy-2) in advanced lung cancer evaluated the effects of combining ganetespib and anti-microtubule agent docetaxel in either KRAS mutant or KRAS wild type non-small cell lung cancer (NSCLC) patients. This trial failed to demonstrate any benefit in terms of progression free survival or overall survival (170). It was then demonstrated that resistance to ganetespib and to the combined treatment with docetaxel in KRAS mutant NSCLC patients was caused by hyperactivation of ERK1/2-p90RSK-mTOR signaling pathway and by circumventing the G2-M checkpoint arrest of the cell cycle (171, 172). These observations suggest that combining ganetespib with ERK1/2, p90RSK, or CDC25C inhibitors could overcome the observed resistance (131). In perspective, one would like to define the biochemical features of the tumor and the fine mechanisms of chaperone/client interaction in order to design HSP90 targeting-molecules that modulate specific signaling hubs, such as transcriptional or epigenetic regulation, maintenance of DNA integrity or bioenergetic circuitries. Such a re-shaping of HSP90 inhibition, aimed at avoiding the deleterious effects of a global damage to cellular proteostasis, would imply selecting different agents for combination usage and reconsidering dosing strategies, adapting them to various cancer patient subsets (173). Novel usage options for HSP90 inhibitors are provided by the combination with targeted therapies or with immune therapy approaches, and preliminary data are encouraging [reviewed in (174)]. Combination of the multi-kinase inhibitor sorafenib with tanespimycin demonstrated efficacy in melanoma and renal cancer patients. HER2-positive, metastatic breast cancer patients treated with tanespimycin showed an improved clinical outcome. Ganetespib induced tumor regression in melanoma xenografts when supplied together with the MEK inhibitor TAK-733 and with the BRAF(V600E) inhibitor vemurafenib. Recently, HSP90 inhibitors were also tested in combination with immunotherapy, and approaches combining inhibitors of immune checkpoints and of HSP90 have been assayed. The anti-PD-L1 antibody STI-A1015 elicited higher therapeutic efficacy in melanoma and colon cancer cell models when combined with ganetespib as compared to the monotherapy regimens. In the case of TRAP1, its genetic inhibition prompts an increase in oxygen consumption rate and a decrease in extracellular acidification rate (78, 95), indicative of a metabolic rewiring toward OXPHOS coupled with a down-modulation of glycolysis. Therefore, it would be promising to identify highly selective TRAP1 inhibitors that do not affect Hsp90 activity. In principle, these compounds could be associated with OXPHOS-targeting molecules (175) in order to induce a bioenergetic catastrophe in tumor cells.

**Table 5:** Preclinical and clinical studies where HSP90 inhibitors were combined with other anti-cancer drugs.

**Hsp90 inhibitor**	**Drugs in combination**	**Cancer type**	**Outcome**	**References**
Tanespimycin (17-AAG)	Sorafenib (multi-kinase inhibitor)	Melanoma, renal and colorectal cancer	Clinical efficacy in melanoma and renal cancer patients	(164)
Tanespimycin	Trastuzumab (anti-HER2 antibody)	HER-2 positive, metastatic breast cancer	Significant anti-cancer activity	(165)
Ganetespib (STA-9090)	Vemurafenib (BRAF(V600E) inhibitor)	Melanoma	Inhibition of tumor growth in BRAF-inhibitor sensitive melanoma	(166)
Ganetespib	TAK-733 (MEK inhibitor)	Melanoma	Tumor regression in vemurafenib-resistant xenografts	(166)
Ganetespib	Capecitabine (DNA synthesis inhibitor)	Colorectal cancer	Improved anti-tumor activity of combinatorial therapy	(167)
Ganetespib	STI-A1015 (anti-PD-L1 antibody)	MC38 colon carcinoma and B16 melanoma	Enhanced anti-tumor efficacy of the combinatorial regimen	(168)
Luminespib (NVP-AUY922)	Omipalisib (PI3K inhibitor)	KRAS mutant NSCLC	Synergistic anti-tumor effect	(169)

Design of new molecules, such as conjugated compounds that link established chemotherapeutic drugs with HSP90 inhibitors, may open further therapeutic windows. This strategy exploits the observation that HSP90 inhibitors highly accumulate in cancer cells, and could therefore act as cargoes for chemotherapeutics, thereby increasing their efficacy while reducing toxicity. The bifunctional compound STA-8666 consists of an HSP90 inhibitor and a topoisomerase inhibitor (SN-38) and demonstrated antitumor effects and lower systemic toxicity in preclinical studies on different cancer models (176, 177). Finally, the simultaneous inhibition of HSP90 and specific protein kinases appears to be another promising avenue to reduce drug resistance (12).

## Conclusions

Understanding the fine details that regulate the function of hub proteins as central as HSP90 and TRAP1 for biochemical and metabolic pathways is a highly challenging task. In this context, chemical biology approaches based on the use of small molecules represent attractive means to expand the reach of our investigations of the complex biology of this chaperone. Designed molecules have the potential to induce variable functional responses by inhibiting or stimulating a certain activity and thus directly focus on the molecular mechanism they are perturbing. In terms of investigation of biological processes, such data must be combined to complementary methods rooted in molecular biology such as pull-downs, genetic screens, CRISPRi, biochemical assays, proteomic and interactomic analyses, expression of mutant proteins where strategically positioned residues are modified. This integrated approach would improve our understanding of the roles of HSP90 in cancer metabolism at different levels. In a more complex scenario, regulation of chaperone networks should also be molecularly investigated during orchestrated responses of the cell to a variety of noxious stimuli such as compartmentalized unfolded protein responses.

Overall, we propose that a molecular understanding of the biology of chaperones through small molecule compounds is important for both fundamental and practical reasons. On the fundamental side, they would illuminate the determinants of biochemical mechanisms. On the practical side, selective chemical tools can expectedly be evolved into compounds that might modulate selected chaperones (and their isoforms) under specific conditions of stress. Finally, combinatorial therapies could aim at simultaneously exposing tumors to specific damaging agents while blunting the activity of protecting chaperones, thus setting the stage for the definition of innovative anti-neoplastic strategies.

## Author Contributions

CS-M, GC, and AR contributed conception and design of the paper. CS-M and SS wrote sections of the manuscript and prepared figures. All authors contributed to manuscript revision, read, and approved the submitted version.

## Conflict of Interest

The authors declare that the research was conducted in the absence of any commercial or financial relationships that could be construed as a potential conflict of interest.
